# Situation analysis of parasitological and entomological indices of onchocerciasis transmission in three drainage basins of the rain forest of South West Cameroon after a decade of ivermectin treatment

**DOI:** 10.1186/s13071-015-0817-2

**Published:** 2015-04-02

**Authors:** Samuel Wanji, Jonas A Kengne-Ouafo, Mathias E Esum, Patrick W N Chounna, Nicholas Tendongfor, Bridget F Adzemye, Joan E E Eyong, Isaac Jato, Fabrice R Datchoua-Poutcheu, Elvis Kah, Peter Enyong, David W Taylor

**Affiliations:** Parasite and Vectors Research Unit, Department of Microbiology and Parasitology, University of Buea, P.O. Box 63, Buea, Cameroon; Research Foundation for Tropical Diseases and Environment, P.O. Box 474, Buea, Cameroon; Tropical Medicine Research station, P.O. Box 55, Kumba, Cameroon; Department of Geography, University of Yaounde1, Yaounde, Cameroon; Department of Biological Sciences, Faculty of Science, University of Bamenda, P.O. Box 39, Bambili, North West Region, Bamenda, Cameroon; Division of Pathway Medicine, School for Biomedical Studies, University of Edinburgh, 49 Little France Crescent, Edinburgh, EH16 4SB UK

**Keywords:** Onchocerciasis, River drainage, Rain forest, Ivermectin, Simulium, Parasitological indices, Entomological indices

## Abstract

**Background:**

Community-Directed Treatment with Ivermectin (CDTI) is the main strategy adopted by the African Programme for Onchocerciasis control (APOC). Recent reports from onchocerciasis endemic areas of savannah zones have demonstrated the feasibility of disease elimination through CDTI. Such information is lacking in rain forest zones. In this study, we investigated the parasitological and entomological indices of onchocerciasis transmission in three drainage basins in the rain forest area of Cameroon [after over a decade of CDTI]. River basins differed in terms of river number and their flow rates; and were characterized by high pre-control prevalence rates (60-98%).

**Methods:**

Nodule palpation and skin snipping were carried out in the study communities to determine the nodule rates, microfilarial prevalences and intensity. *Simulium* flies were caught at capture points and dissected to determine the biting, parous, infection and infective rates and the transmission potential.

**Results:**

The highest mean microfilaria (mf) prevalence was recorded in the Meme (52.7%), followed by Mungo (41.0%) and Manyu drainage basin (33.0%). The same trend was seen with nodule prevalence between the drainage basins. Twenty-three (23/39) communities (among which 13 in the Meme) still had mf prevalence above 40%. All the communities surveyed had community microfilarial loads (CMFL) below 10 mf/skin snip (ss). The infection was more intense in the Mungo and Meme. The intensity of infection was still high in younger individuals and children less than 10 years of age. Transmission potentials as high as 1211.7 infective larvae/person/month were found in some of the study communities. Entomological indices followed the same trend as the parasitological indices in the three river basins with the Meme having the highest values.

**Conclusion:**

When compared with pre-control data, results of the present study show that after over a decade of CDTI, the burden of onchocerciasis has reduced. However, transmission is still going on in this study site where loiasis and onchocerciasis are co-endemic and where ecological factors strongly favour the onchocerciasis transmission. The possible reasons for this persistent and differential transmission despite over a decade of control efforts using ivermectin are discussed.

**Electronic supplementary material:**

The online version of this article (doi:10.1186/s13071-015-0817-2) contains supplementary material, which is available to authorized users.

## Background

Onchocerciasis, or river blindness, is a chronic disease caused by infection with *Onchocerca volvulus*. It is characterized by the presence of subcutaneous nodules harbouring adult parasites and presentation of a dermatitis that can be extremely severe, visual impairment and in some cases blindness. It has been estimated that 36 million people are infected [1] and 86 million people live in high risk areas in the APOC countries [2,3].

Onchocerciasis control strategies have evolved over the years from isolated and small scale vector control operations in West Africa [4, (Walsh JF: The control of Simulum damnosum in the River Niger and its tributaries in relation to the Kanji Lake Research project. Covering the period 1961–1969. WHO unpublished mimeographed document PD 70.4, 1970)] to the regional Onchocerciasis Control Programme (OCP) launched in 1974. The OCP successfully interrupted onchocerciasis transmission and ultimately eliminated the disease as a public health problem from savannah areas of 11 participating West African countries through use of aerial larviciding of vector breeding sites. However, this method of control was considered neither feasible nor cost effective in the forest regions of Africa where over 85% of the 36 million infected people live [5].

The fight against onchocerciasis was revolutionized with the introduction in 1987 of ivermectin (Mectizan®) for treatment of the disease. A single annual dose of ivermectin can clear the skin of microfilariae and consequently reduce morbidity associated with the infection. The drug is safe and only a few minor side effects have been reported, which is in contrast to diethylcarbamazine (DEC) the drug previously used to treat onchocerciasis [6,7]. The availability of ivermectin facilitated the creation of the African Programme for Onchocerciasis Control (APOC) that extends treatment to all the remaining onchocerciasis endemic areas in Africa [8]. APOC’s initial main goal was to support the establishment of Community-Directed Treatment with ivermectin (CDTI) [9] with the strategic objective to reduce prevalence and transmission of onchocerciasis to a point where the disease will no longer be a public health problem in African countries not previously covered by the OCP. This was to be achieved through a sustained delivery of an annual dose of ivermectin for a period of at least 15 years with a minimum treatment coverage rate of 65% in the communities. Ivermectin is manufactured by Merck & Co., Inc (New Jersey, USA) who have agreed to donate the drug free-of-charge for as long as required to achieve this strategic objective.

After 10 to 17 years of ivermectin treatment, evaluation conducted in some CDTI projects have reported prevalence rates and microfilarial loads as well as transmission indices below the thresholds required for elimination [10-13]. These reports came from CDTI projects situated in savannah regions. However, there is a paucity of such information from rain forest areas where conditions are much more favourable for transmission. Here, presence of perennial fast flowing rivers favours the breeding and development of black flies that contributes to transmission. Furthermore, in many forest regions CDTI programmes can be compromised by the presence of *Loa loa* by triggering severe adverse reactions in high microfilaraemic individuals following ivermectin treatment [14-16].

This study was designed to determine the parasitological and entomological indices of onchocerciasis transmission after 10 to 12 years of mass treatment with ivermectin in three drainage basins with contrasting hydrologic profiles in the rain forest areas co-endemic for onchocerciasis and loiasis in Cameroon. Our findings are compared with both historical data (when available) and the trends of changes predicted by ONCHOSIM mathematical model.

## Methods

### Study design

This was a cross-sectional study designed to assess onchocerciasis prevalence, intensity and entomological indices of transmission in 3 contrasting hydrographical basins in the rain forest of Cameroon. These river basins are different in terms of geography and topography. The sources of the main rivers are different and the topography of the environment confers different river flow rates in the various river basins and by so doing confers variable conditions for *Simulium* breeding. Moreover, in the Manyu, the communities are situated in the valleys where river flow rates are lower compared to those found in the Mungo and Meme where communities are situated on the slopes. A total of 39 communities were selected for the study among which 11, 12 and 16 were found in the Manyu, Mungo and Meme river basins respectively. The majority (36/39) of the communities were under community-directed treatment with ivermectin (CDTI) while 3 (precisely in the Manyu river basin) employed Clinic-Based Treatment with Ivermectin (CBTI). In these communties, selective treatment was carried out by the health personnel based on the *Loa* microfilaremia levels of the individuals. Briefly, a preliminary diagnosis was carried out before treatment and individuals with high *Loa loa* microfilaraemia who presented high risk of severe adverse events following ivermectin treatment were consistently excluded from the treatment. The study area is covered by two CDTI projects; South west I CDTI project operating within the Mungo and Meme hydrographical basins and South west II covering the Manyu river basin). Treatment had been going on for more than a decade (10–12 years) with geographical coverage varying between 95-100% and therapeutic coverage generally above 65%. Some cases of severe adverse reactions had been reported in this area at the onset of ivermectin treatment and in other CDTI projects in Cameroon [17-20]. Data on the CDTI therapeutic coverage (1999–2009) obtained from the regional onchocerciasis control programme, South west region of Cameroon indicate low coverage (<50%) at the onset of both CDTI projects (1999–2003). This value gradually went up to 84% between 2004 and 2009.

Study participants comprise individuals of both sexes aged 5 years and above. Parasitological and entomological surveys were carried out in the months of April and July in 2011 and 2012 during which the following indices of onchocerciasis were generated: Mf prevalence, nodule prevalence, Mf intensity (CMFL/WMMfD), age influence (children/young [5–14 years] and adults [>14 years] individuals) on the Mf prevalence and intensity, sex influence on the Mf prevalence and intensity. In calculating the CMFL, only individuals aged > 20 years were considered while the Williams mean mf density (WMMfD) was used to express the intensity of infection in age and sex stratified populations. Entomological indices of onchocerciasis transmission included *Simulium* biting, infection and infective rates, number of L3s per 1000 parous flies and monthly transmission potential (L3/man/month).

The parasitological and entomological indicators of onchocerciasis transmission generated from this study were compared to 1) historical data (in communities where these exist); 2) to the trends of changes predicted by ONCHOSIM mathematical model, taking into consideration the pre-treatment level of endemicity of onchocerciasis and treatment coverage in the study area. Historical data were obtained both from published research articles and results from unpublished works carried out by our research team using the same protocol as the one described in the present study [21,22]. The comparisons between pre-control and post-control results were done on data collected during the same months.

A total of 7 *Simulium* collection points (2, 2 and 3 in the Manyu, Mungo and Meme drainage basins respectively) were selected for the study. Three collection points were selected in the Meme drainage basin because the majority of the study communities (16) were found in this basin (Additional file 1: Figure S1). Some of the communities had a small population hence the disparity in the sample size per community.

### Ethical considerations

Prior to recruitment, the nature and objectives of the study were explained to potential participants and those who agreed to take part in the study signed a consent form while an assent was obtained from parents or guardians of children who were enrolled in the study. Participation was voluntary. All volunteers were handled in accordance with the Helsinki declaration on the use of humans in biomedical research. This study was approved by the Cameroon Ethics Committee and the Ministry of Public Health. Fly collectors were given a dose of ivermectin as prophylactic treatment against filariasis before the entomological survey.

### Study site

**Figure 1:**
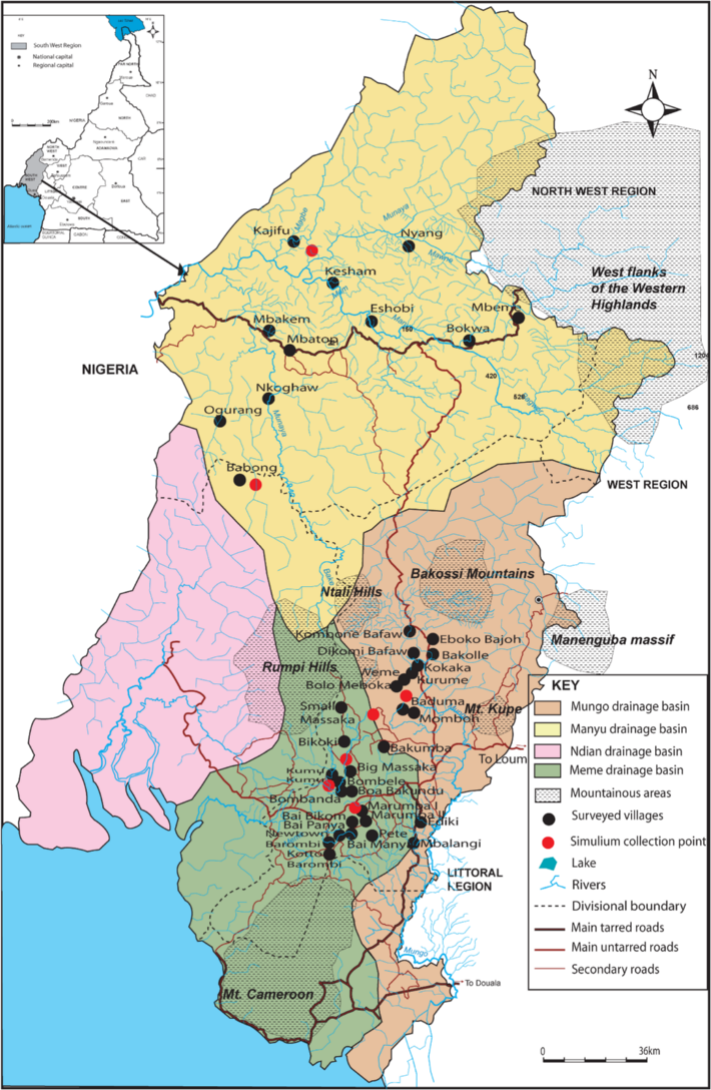
**Distribution of the study communities and Simulium collection sites in the three river drainages.**

Study sites were selected from three hydrographic basins namely Manyu, Mungo and Meme, all situated in the rain forest in the South West region of Cameroon approximately 60 km from the Atlantic Ocean. The topography is very diverse; the main feature being a mountain range (Rumpi Hills, Ntali Hills, Bakossi mountain, Mount Manenguba) characterized by a volcanic ridge culminating at 1764 metres with a northeast orientation [23]. The volcanic ridge is broken by several valleys and constitutes a watershed from which several rivers (Munaya, Meme, Mungo, Ndian) take their source (Figure 1). These rivers go down steep slopes generating fast currents and appropriate flow rates that favour the establishment of permanent *Simulium* breeding sites conducive for continuous onchocerciasis transmission. The climate is characterized by 8 months of rainfall and a short dry season from December to March. The annual rainfall varies between 2500 to 4000 mm with annual temperatures ranging from 25 to 32°C. The vegetation is dense evergreen and humid rain forest that is gradually being degraded for lumbering and agricultural activities. In the Manyu river basin, the population consists mainly of the Bayangs and Anyangs while in the Mungo and Meme river basin, there is a predominance of the Bakundus, Bafaws, Mbonges and immigrants from other parts of Cameroon and Nigeria. The area is endemic for loiasis with mf prevalence rates ranging from 6.3% to 23.1% in individuals aged 15 years and above [14,24,25].

### The Manyu hydrographic basin

This basin is composed of rivers flowing down the western flank of the main mountain ridge. The main river in this forest area is the Manyu with the main tributaries being the Mam, Munaya North and Munaya South. In the North, the tributaries of Munaya constitute important *Simulium* breeding sites in communities such as Mbakem, Kajifu and Nyang. The tributaries of the Mam River form breeding sites in Kesham while sub-tributaries of the Manyu constitute the rivers in Eshobi. In the South, the rivers are tributaries of both the Manyu and the Munaya and form breeding points in most of the communities under CBTI. The Munaya, Mam and Manyu flow into the Cross river. Of the eleven communities selected in the Manyu drainage basin, 3 namely (Babong, Nkoghaw, Oguran) were under CBTI. They were under CBTI because the area is co-endemic with loasis. The initial Rapid Assessment Procedure for Loaisis (RAPLOA) results revealed prevalences as high as 60% and more which was an indication that the risk to develop SAEs following mass drug administration in the area was too high [14,26].

### The Mungo hydrographic basin

The main river (Mungo River) takes its source in the Rumpi hills with tributaries from the Mount Manenguba and Ntali hills. The Mungo River flows some two kilometers from selected villages and its tributaries constitute numerous breeding sites for the *Simulium* flies (Kumba River, Kendongi River, Dilolo and Menge). The lowest flow rate observed is 16.5 m^3^/s in January and the highest flow rate is 950 m^3^/s in September [27]. Twelve Communities were selected in this basin.

### Meme river basin

The River Meme (Main River) and its tributaries originate from the Rumpi hills south west of the main mountain range and flow down precipitous slopes some kilometers from the selected communities. The main tributaries of the Meme river are the Bile river which goes pass Marumba I and II, the Uve river in Bakumba and Big Massaka and Meme in Bombele. These tributaries create important *Simulium* breeding sites as they enter the Meme River. The rivers become extremely fast reaching 800 m^3^/s after a heavy rain downpour but low flow rates of approximate 9.1 m^3^/s. can be observed during the dry season. The highest river flow rates are found in this river basin followed by the Mungo and then the Manyu. The flat-like nature of Manyu river basin contributes to the establishment of low flow rates observed. Sixteen communities were selected in the Meme hydrographic basin.

### Parasitological evaluation

Nodule palpation and skin snipping were carried out to determine the presence of the parasite. This was done according to a method described by previous authors [28,29]. Before parasitological examination, participants’socio-demographic data such as name, sex, age, occupation and duration of stay in the community were collected using a structured questionnaire.

### Nodule palpation

Participants who gave their consent were examined individually in a well-lit private room. Clinical examinations were performed on the partially undressed participants, paying attention to bony prominences of the torso, iliac crest and upper trochanter, arms and legs. Participants were examined for the presence of nodules and nodule prevalence was expressed according to Ngoumou *et al.* [30].

### Skin snipping

After the clinical examination, two skin biopsies from the posterior iliac crest were taken using a 2 mm corneo-scleral punch (CT 016 Everhards 2218–15 C, Germany). The skin samples from each participant were placed in two separate wells of a microtitre plate containing 2 drops of sterile normal saline. The corresponding well numbers were reflected on the participant’s form. The plates were sealed with parafilm to prevent any spill over or evaporation and incubated at room temperature for 24 hours [28,29]. All emerged mf were counted using an inverted microscope (Motic AE21) at X10 magnification and expressed per skin snip.

### Entomological evaluation

#### Capture of wild *Simulium* flies

Fly collection took place in the month of July 2012. The fly collection team at each site was composed of two trained individuals, one working between 07:00 and 12:00 hours and the other between 12:00 and 18:00 hours for 5 days except in Kajifu (Manyu drainage basin) site where flies could not be collected on the 5th day due to heavy rainfall. Female *Simulium* flies coming to their exposed legs for a blood meal were captured using suction tubes or mouth aspirators before they bite. Flies were collected by the same work force at all stations (collection points) during the entire study [21,22].

#### Dissection of *Simulium* flies

Captured flies were killed using chloroform, counted and dissected in physiological saline under a dissecting microscope. Flies caught were recorded and dissected on an hourly basis to determine their parity and infection. The dissection technique consisted of holding the fly with a needle in the thorax, piercing the abdomen with a dissecting needle at the posterior end and then pulling out the different internal organs to examine the quantity of fat bodies, the state of the malpighian tubules and the ovaries in order to distinguish parous from nulliparous flies. The head, thorax and abdomen of parous flies were further dissected separately and examined for *Onchocerca volvulus* developing larvae (L1, L2 and L3). Any infections found were counted and recorded on a dissection sheet [21].

### Data analysis

All the data generated were keyed in Epi info 6 and analyzed using SPSS version 20. The mf prevalence was expressed as a percentage (number of persons positive for mf divided by number examined × 100). Intensities of infection in the communities were assessed as the CMFL, the reference index used in the OCP, as the geometric mean of individual mf loads. The calculation was done using the log(X +1) transformation, where x is the individual microfilaria load [30]. However, WMMfD was used for stratified populations. The transformed data were subjected to t-test and analysis of variance (ANOVA) to determine the significant differences in WMMfD/CMFL between males and females, the different age groups and communities. Chi-square test was used to check for significant differences in mf and nodule prevalence between communities and; males and females. The same test was also used to compare pre-treatment/control and post-treatment parasitological and entomological indicators. To ensure adequate comparisons in time and space, nodule and mf prevalence were gender- and age-adjusted using the WHO/OCP standardization scale [31] previously modified by Boussinesq *et al.* [32]. The modified standardization scale was used because study participants were all aged five years and above. All the tests were performed at the 5% level of significance.

The data generated from fly collections and dissections were used in the calculation of entomological indices as per the standard methodology.

Monthly biting rate (MBR) = (number of flies captured × number of days in the month)/number of fly collection days [21].

Monthly transmission potential (MTP) = (number of days in the month × number of infective (L3) larvae)/number of days worked × (number of flies collected/number of flies dissected) [21].

## Results

### Study communities and population

A total of 39 communities were surveyed. 2797 individuals took part in the study with a mean age of 35.86 years (age range 5–95). Out of this number, 761 (356 males and 405 females) were examined in the Manyu drainage basin, 995 (536 males and 459 females) in the Mungo and 1041(578 males and 463 females) in the Meme. The Manyu drainage basin population was composed of 124 children (5–14 years) and 637 adults (>20 years), in the Mungo it was made up of 298 children and 697 adults and 964 adults in the Meme. No child was examined in the Meme river basin. The adult study participants were mainly farmers (98%), involved in the production of cocoa, palm oil, plantains, and cocoyam.

### Parasitological and entomological indices

#### The Manyu drainage basin

**Figure 2:**
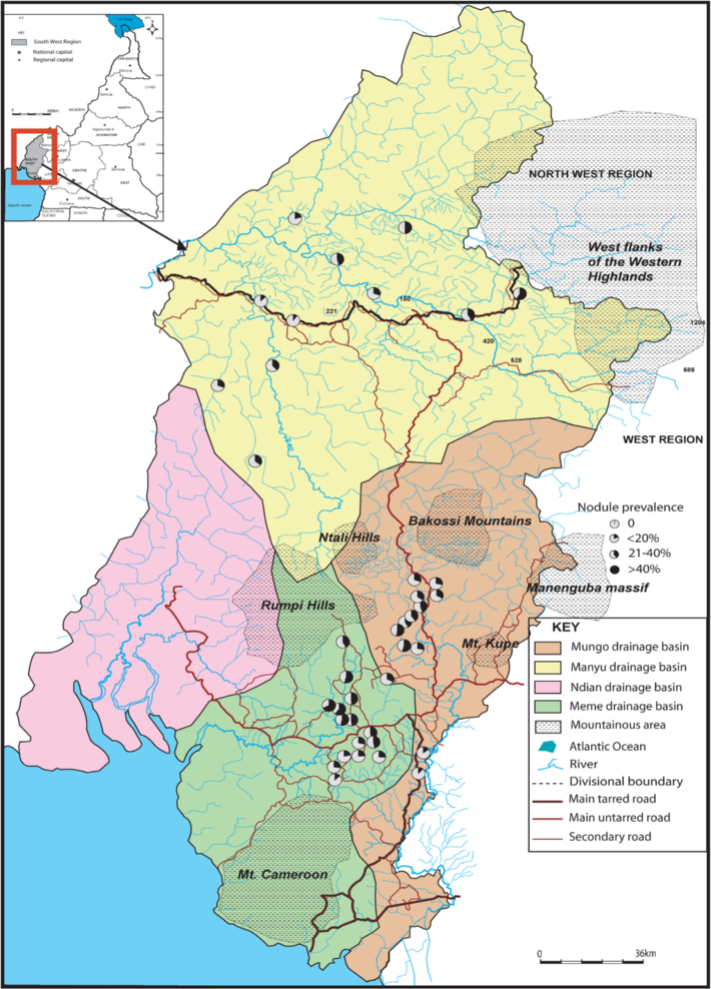
**Nodule prevalence in the three drainage basins.** (The pie is 100% black when nodule prevalence is 100%).

**Figure 3:**
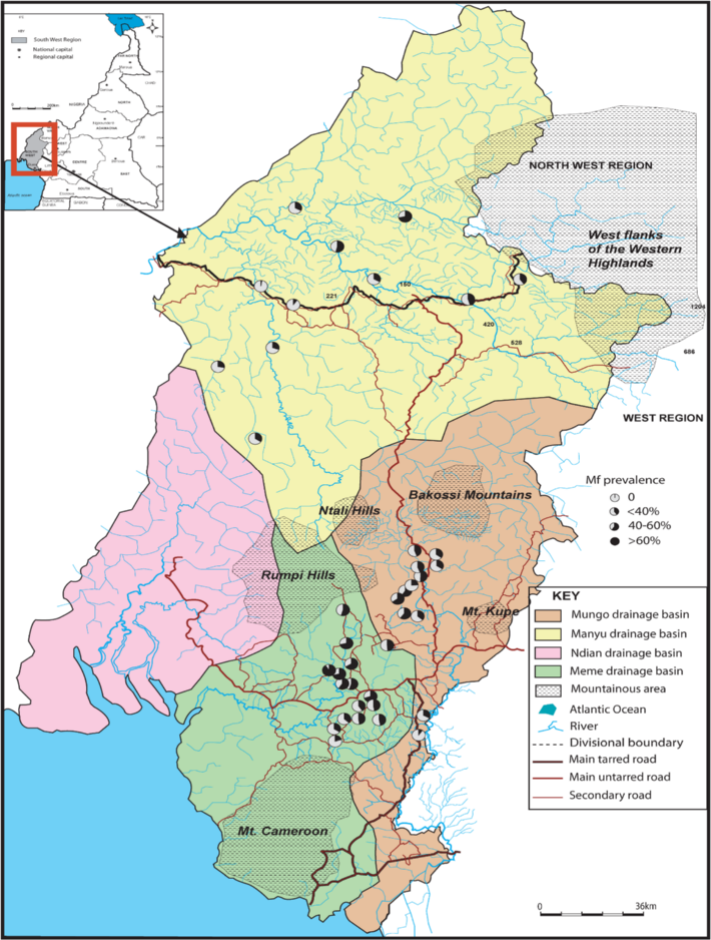
**Mf prevalence in the three drainage basins.** (The pie is 100% black when mf prevalence is 100%).

**Figure 4:**
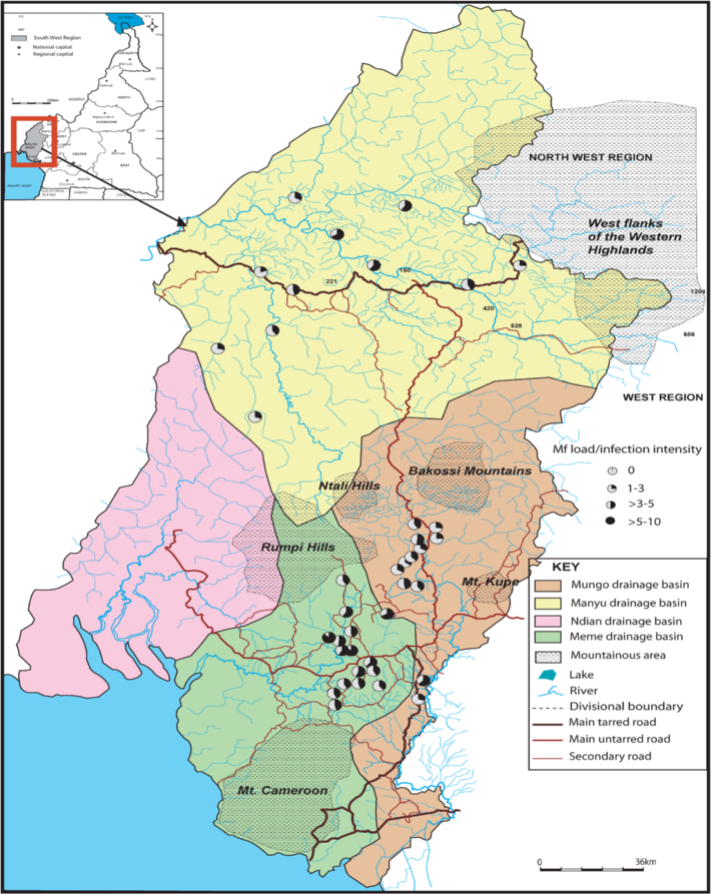
**CMFL in the three drainage basins.** (The pie is 100% black when the CMFL is 10 mf/snip).

The overall raw nodule and Mf prevalence was 33.0% for each. Nodule prevalence (both raw and adjusted) varied from 10% to 58.82% (P < 0.001). Nine communities had nodule prevalence greater than 20% with 3/11 above 40% (Table 1 and Figure 2). The number of communities with nodule prevalence > 40% increased by 1 with adjustment. Mf prevalence ranged from 2.5% to 71.87% though up to 83.0% was obtained when adjusted (P < 0.001). Three out of 11 communities had raw mf prevalence greater than 40% against 4/11 when adjusted (Table 1 and Figure 3). The overall CMFL was 3.65 mf/ss in the Manyu drainage basin. All the communities had CMFL below 10 mf/ss. CMFL also varied in the communities, from 2.0 mf/ss to 6.74 mf/ss (P < 0.001, Table 1, Figure 4).

**Table 1:** **Raw and gender-and age-adjusted onchocercal nodule and microfilarial prevalence and intensity in the Manyu, Mungo and Meme drainage basins**

**Drainage basin**	**Villages**	**Latitude**	**Longitude**	**Nb examined**	**Nb > 20 years**	**Raw mf prevalence**	**Adjusted Mf prevalence**	**Raw nodule prevalence**	**Adjusted nodule prevalence**	**CMFL (Mf/ss)**
MANYU	Bokwa	N05.71666°	E09.63333°	60	35	27(45.0)	54.8	25(41.6)	44.7	4.2
	Eshobi	N05.78333°	E09.36666°	90	85	30(33.3)	34.4	27(30)	19.9	6
	Kajifu	N05.09666°	E09.18333°	101	75	34(32.6)	35.2	21(20.8)	16.8	3.2
	Kesham	N05.86666°	E09.28333°	39	23	21(53.8)	56.9	18(46.1)	50.9	6.7
	Mbakem	N05.73338°	E09.09669°	39	37	1(2.5)	3.1	5(12.8)	13.8	2
	Mbatop	N05.71913°	E09.11888°	80	62	8(10.0)	12.1	8(10)	10.6	4.6
	Mbeme	N05.76666°	E09.76666°	85	80	34(40.0)	41.8	50(58.8)	58.2	2.5
	Nyang	N05.95000°	E09.41666°	32	19	23(71.8)	83	16(50)	46	5.9
	Ogurang	N05.46666°	E08.95000°	82	62	23(28.1)	28.6	25(30.5)	24.6	2.8
	Babong	N05.05000°	E09.05000°	93	82	32(34.4)	32.4	33(35.5)	35.3	3
	Nkoghaw	N05.56666°	E09.10000°	60	45	18(30.0)	31	23(38.3)	32.2	3.9
	**Total**			**761**	**605**	**251(33.0)**	**33.8**	**251(33)**	**28.2**	**3.6**
MUNGO	Baduma	N04.83333°	E09.43333°	70	47	42(60)	62.1	38(54.2)	51.2	4.6
	Bakolle	N04.96666°	E09.51666°	78	37	27(34.6)	32.3	26(33.3)	35.6	2.6
	Bolo	N04.86666°	E09.43333°	95	50	62(65.2)	62.6	50(52.6)	50.9	3.3
	Dikomi-Bafaw	N04.96666°	E09.46666°	100	62	44(44)	41.7	34(34)	39.6	5.2
	Eboko-Bajor	N04.98333°	E09.51666°	88	61	31(35.2)	33.7	25(28.4)	29.4	2.8
	Ediki	N04.54441°	E09.46444°	101	75	33(32.6)	34.3	15(14.8)	20.3	6.3
	Kokaka	N04.91666°	E09.46666°	73	44	38(50.6)	56	33(45.2)	42.5	3.2
	Kombone-Bafaw	N05.00000°	E09.45000°	97	60	43(44.3)	43.2	30(30.9)	31.9	4.1
	Kurume	N04.90000°	E09.45000°	45	28	11(24.4)	39.6	18(40)	35.3	3.7
	Mbalangui	N04.49750°	E09.45997°	101	85	10(9.9)	11.8	12(11.8)	13.1	2.9
	Momboh	N04.81666°	E09.46666°	88	57	32(36.3)	39.5	25(28.4)	35.2	3.9
	Weme	N04.88333°	E09.43333°	59	28	35(59.3)	46.1	25(42.3)	58.9	3.4
	**Total**			**995**	**634**	**408(41)**	**42.5**	**331(33.2)**	**32.7**	**3.7**
MEME	Bai-Bikom	N04.55092°	E09.33332°	91	88	37(40.6)	48.3	26(28.5)	25.5	5.6
	Bai-Manya	N04.53364°	E09.32434°	89	85	43(48.3)	52.4	22(24.7)	25	4.4
	Bai-Panya	N04.52274°	E09.29944°	43	41	16(37.2)	34.7	9(20.9)	17.7	4.2
	Bakumba	N04.77192°	E09.28150°	76	75	37(48.6)	50.7	25(32.8)	25.6	6.7
	Big-Massaka	N04.68737°	E09.29251°	157	156	104(66.2)	68.3	77(49.1)	47.9	4.8
	Bikoki	N04.74523°	E09.28374°	46	42	33(71.7)	49.2	25(54.3)	44.7	5.9
	Boa-Bakundu	N04.62503°	E09.29812°	60	59	33(55)	64.3	29(48.3)	56	8.3
	Bombanda	N04.63250°	E09.27426°	32	30	19(59.3)	64.2	17(53.1)	51.2	5.8
	Bombele	N04.64910°	E09.26020°	66	56	47(71.2)	74	40(60.6)	60.6	5.2
	Kotto-Barombi	N04.46825°	E09.25617°	66	65	14(21.2)	28.6	9(13.6)	15	4.6
	Kumu-Kumu	N04.65166°	E09.24125°	34	25	31(91.1)	89.2	23(67.6)	77.4	8.7
	Marumba I	N04.58773°	E09.34178°	50	46	35(70)	73.7	24(48)	51.1	6.1
	Marumba II	N04.57106°	E09.34343°	68	67	33(48.5)	52.2	32(47)	47	4.1
	Newtown-Barombi	N04.49959°	E09.28003°	96	95	33(34.3)	32.2	26(27)	22.1	4.2
	Pete-Bakundu	N04.53776°	E09.36548°	34	33	16(47.1)	62.3	9(26.4)	40.4	3.9
	Small-Massaka	N04.80318°	E09.28325°	33	33	18(54.5)	53	14(42.4)	31.6	4
	**Total**			**1041**	**996**	**549(52.7)**	**54.3**	**407(39.1)**	**36.2**	**5.2**

Nb = Number; CMFL = Community Microfilarial Load, Mf = Mirofilaria, Number > 20 yrs was used in calculating the CMFL.

The total line (bold) indicates the overall number of individuals examined, overall mf and nodule prevalence; and the overall intensity of infection for each drainage basin.

As depicted in Table 2, the prevalence was significantly higher in male than female subjects both for nodules (P < 0.001) and mfs (P = 0.044). WMMfD was also slightly higher for male (4.07 mf/ss) than female (3.54 mf/ss) subjects (P = 0.163).

**Table 2:** **Raw microfilaria, nodule prevalence and** ***WMMfD*** **in the three drainage basins by sex and age**

		**Number examined**		**Mf prevalence**			**Nodule prevalence**			**WMMfD(mf/ss)**		
**River basin**	**Age group**	**Male**	**Female**	**Male**	**Female**	**Overall**	**Male**	**Female**	**Overall**	**Male**	**Female**	**Overall**
MANYU	[5-9]	33	28	8(24.2)	8(28.6)	26.2	8(24.2)	6(21.4)	23	6.71	5.81	6.23
	[10-14]	37	26	17(45.9)	2(7.7)	30.2	7(18.9)	1(3.8)	12.7	5.26	3.54	5.03
	**Children**	**70**	**54**	**25(35.7)**	**10(18.5)**	**28.2**	**15(21.4)**	**7(13)**	**17.7**	**5.65**	**5.19**	**5.51**
	[15-29]	54	114	28(51.9)	38(33.3)	39.3	24(44.4)	16(14)	23.8	3.09	3.89	3.51
	[30-49]	57	93	22(38.6)	30(32.3)	34.7	25(43.9)	24(25.8)	32.7	3.54	3.58	3.57
	>50	175	144	55(31.4)	42(29.2)	30.4	83(47.4)	57(39.6)	43.9	4.41	3.02	3.69
	**Adults**	**286**	**351**	**105(36.7)**	**110(31.3)**	**33.8**	**132(46.2)**	**97(27.6)**	**35.9**	**3.8**	**3.43**	**3.6**
	**Total**	**356**	**405**	**130(36.5)**	**120(29.6)**	**33.0**	**147(41.3)**	**104(25.7)**	**33.0**	**4.07**	**3.54**	**3.8**
MUNGO	[5-9]	74	60	15(20.3)	32(53.3)	35.1	34(45.9)	20(33.3)	40.3	3.64	7.03	5.53
	[10-14]	89	75	28(31.5)	45(60)	44.5	21(23.6)	13(17.3)	20.7	4.89	4.69	4.77
	**Children**	**163**	**135**	**43(26.4)**	**77(57)**	**40.3**	**55(33.7)**	**33(24.4)**	**29.5**	**4.38**	**5.48**	**5.04**
	[15-29]	125	112	42(33.6)	72(64.3)	48.1	47(37.6)	29(25.9)	32.1	5.3	3.65	4.14
	[30-49]	79	60	15(19)	35(58.3)	36	29(36.7)	20(33.3)	35.3	5.16	3.12	3.56
	>50	169	152	43(25.4)	80(52.6)	38.3	75(44.4)	43(28.3)	36.8	3.52	3.82	3.71
	**Adults**	**373**	**324**	**100(26.8)**	**187(57.7)**	**41.2**	**151(40.5)**	**92(28.4)**	**34.9**	**4.36**	**3.61**	**3.84**
	**Total**	**536**	**459**	**143(26.7)**	**264(57.5)**	**41.0**	**206(38.4)**	**125(27.2)**	**33.3**	**4.37**	**4.03**	**4.14**
MEME	**Children**	**-**	**-**	**-**	**-**	**-**	**-**	**-**	**-**	**-**	**-**	**-**
	[15-29]	179	146	114(63.7)	90(61.6)	62.8	69(38.5)	43(29.5)	34.5	8.73	4.96	6.66
	[30-49]	145	93	77(53.1)	43(46.2)	50.4	60(41.4)	27(29)	36.6	5.51	4.49	5.11
	>50	254	224	113(44.5)	112(50)	47.1	106(41.7)	102(45.5)	43.5	4.86	4.31	4.57
	**Adults**	**578**	**463**	**304(52.6)**	**245(52.9)**	**52.7**	**235(40.7)**	**172(37.1)**	**39.1**	**6.13**	**4.57**	**5.34**
	**Total**	**578**	**463**	**304(52.6)**	**245(52.9)**	**52.7**	**235(40.7)**	**172(37.1)**	**39.1**	**6.13**	**4.57**	**5.34**

NB: The numbers in brackets represent prevalences.

Bold data indicate the mean mf and nodule prevalence; and mean intensity of infection in children and adults for each river basin.

The total line (bold) indicates the overall mf and nodule prevalence and intensity of infection for each river basin.

Nodule prevalence was lower in younger individuals under 14 years (17.7%) than in those above 14 years of age (35.9%). This difference was statistically significant (P < 0.0001, Table 2). Mf prevalence was also lower in children (28.2%) than adults (33.8%) but the difference was not significant (P = 0.231).

Taking into account the disparity in the number of males and females, number of participants in the various age groups between the three drainage basins and the fact that the generated data (prevalences) were to be compared to historical data and between the river basins, (Table 2), nodule and mf gender- and age-adjusted prevalences were also calculated. The latter showed the same trend observed with raw prevalences with male subjects being more infected than female counterparts (Table 3) and children having lower prevalence than adults (Table 4). The intensity of infection was significantly higher in younger individuals than in adults (P = 0.034; Table 2). The same trend was observed when splitting the two age groups into smaller ones with the infection being more intense in children ≤ 10 years (Table 2).

**Table 3:** **Raw and gender- and age-adjusted mf and nodule prevalence in the three drainage basins by sex**

**Drainage basin**	**Gender**	**Raw mf prevalence**	**Adjusted mf prevalence**	**Raw nodule prevalence**	**Adjusted nodule prevalence**
**Manyu**	Male	36.5	39.5	41.3	36.0
	Female	29.6	28.0	25.7	20.4
	**Overall**	**33**	**33.8**	**33**	**28.2**
**Mungo**	Male	26.7	25.9	38.4	36.9
	Female	57.5	59.1	27.2	28.4
	**Overall**	**41.0**	**42.5**	**33.3**	**32.7**
**Meme**	Male	52.6	55.3	40.7	40.0
	Female	52.9	53.4	37.1	32.5
	**Overall**	**52.7**	**54.3**	**38.1**	**36.2**

NB: Bold data represent overall mf and nodule prevalence (both raw and adjusted) for each drainage basin.

**Table 4:** **Raw and gender-and age-adjusted mf and nodule prevalence in the three drainage basins by age**

		**Drainage basin**					
		**Manyu**		**Mungo**		**Meme**	
	**Age group**	**Raw**	**Adjusted**	**Raw**	**Adjusted**	**Raw**	**Adjusted**
**Mf prevalence**	**Children**	28.2	26.5	40.3	41.3	-	-
	**Adults**	33.8	37.3	41.2	43.1	52.7	54.3
**Nodule prevalence**	**Children**	17.7	17.1	29.5	30.1	-	-
	**Adults**	35.9	34.1	34.9	34.0	39.1	36.2

The results of *Simulium* dissection from the 2 catching points of the Manyu drainage basin are presented in Table 5. The number of L3s found in the head of dissected flies was 60.7 and 88.8 L3/1000 parous flies for the collection sites of Babong and Kajifu respectively.

**Table 5:** **Capture and dissection of** ***Simulium squamosum*** **from different drainage basins**

**Drainage basin**	**Manyu**		**Mungo**		**Meme**		
**Entomological indices**	**Kajifu**	**Babong**	**Bolo**	**Bakumba**	**Big Massaka**	**Bombele**	**Marumba I**
Females captured	4051	5683	2106	4068	1917	3119	1015
Daily biting rate	1012.8	1623.7	421.2	813.6	383.4	623.8	203
Monthly biting rate	30382.5	48711.4	12636	24408	11502	18714	6090
Females dissected	4051	4040	2106	3961	1862	3119	1015
Parous females	428	519	588	1058	767	908	493
Infected females (L1,L2,L3)	57	19	49	114	112	117	21
% infected females	13.3	3.7	8.3	10.8	14.6	12.9	4.3
Females with L3 in head	12	9	16	64	49	48	10
% infective females	2.8	1.7	2.7	6.0	6.4	5.3	2.0
L3/infective female	3.2	2.88	2.9	3.2	4.2	3.9	1.5
L3 in head/1000 parous	88.8	60.7	79.9	190.9	271.2	207	30.4
MTP	285	313.5	282	1180.1	1211.7	1128	90

**Figure 5:**
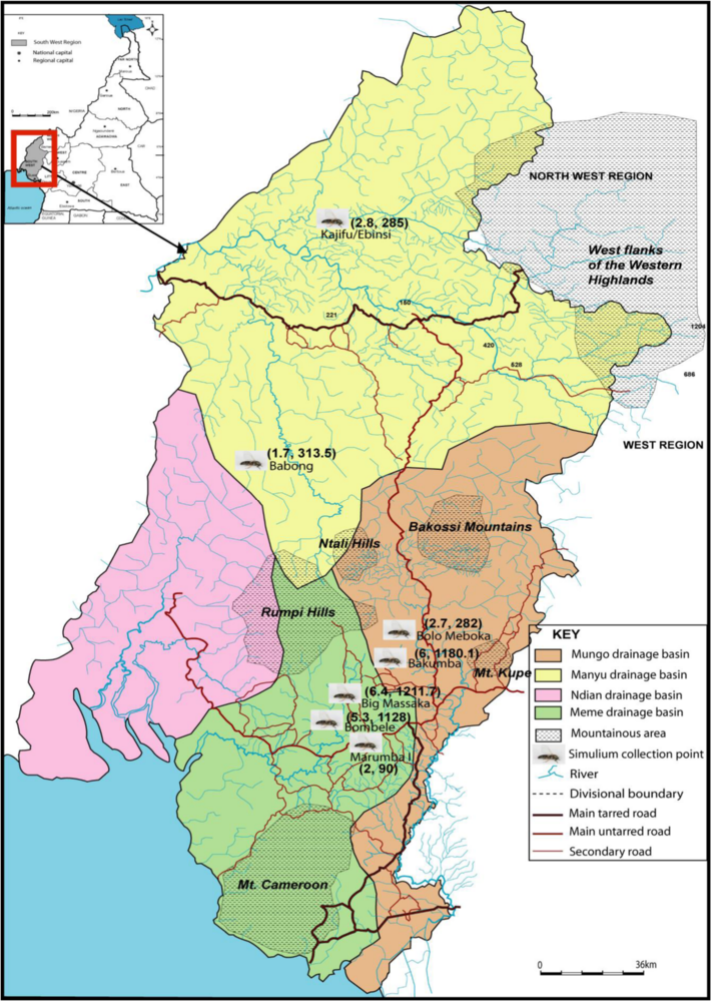
**Simulium collection points and onchocerciasis transmission indices in the three drainage basins.** (On the map in brackets are the infective rates and monthly transmission potentials for each collection point).

The transmission potentials were 313.5 and 285 infective larvae/man/month for Babong and Kajifu respectively. The infective rates were 1.7 and 2.8% at Babong and Kajifu respectively (Tables 5 and 6; Figure 5).

**Table 6:** **Monthly biting rate, infective rates and monthly transmission potentials at different sites in the study area**

**Capture site**	**River**	**Latitude**	**Longitude**	**MBR**	**Infective rate (%)**	**MTP**	**Drainage basin**
Kajifu/Ebinsi	Ebinsi	N05.93333°	E09.21666°	30382.5	2.8	285	Manyu
Babong	Banks (Monaya River)	N05.05000°	E09.05000°	48711.4	1.7	313.5	
Bolo	Dilolo	N04.86666°	E09.43333°	12636	2.7	282	Mungo
Bakumba Bridge	Uve	N04.76666°	E09.31666°	24408	6	1180.1	
Bombele	Meme	N04.64910°	E009.26020°	18714	5.3	1128	Meme
Big Massaka	Uve	N04.68737°	E009.29251°	11502	6.4	1211.7	
Marumba I	Bile	N04.58773°	E009.34178°	6090	2	90	

(Flies/man/month) = Monthly biting rate.

MTP (L3/man/month) = Monthly transmission potential.

#### Mungo drainage basin

In this basin, 331 (33.26%) were positive for nodules and 408 (41.00%) for mfs (Table 1). Nodule prevalence ranged from 11.88% to 54.28% for raw (P < 0.001) and 13.1% to 58.9% for adjusted prevalence. Ten (10) out of 12 communities had raw nodule prevalence greater than 20% with 5/12 above 40% (Table 1 and Figure 2). These figures changed with adjusted prevalence (11 and 4 communities with > 20% and > 40% adjusted nodule prevalence respectively). Raw mf prevalence ranged from 9.90% to 65.26% (P < 0.001). Six out of 12 communities had mf prevalence greater than 40% with both raw and adjusted prevalence (Table 1 and Figure 3). The overall CMFL was 3.78 mf/ss relatively higher than that found in the Manyu drainage basin (P = 0.98). All the communities had CMFL below 10 mf/ss. CMFL also varied in the communities, from 2.79 to 6.31 mf/ss (P = 0.3, Figure 4, Table 1). The mf prevalence was higher than the one in the Manyu river basin (P = 0.0007) while nodule prevalence was similar in the two basins (P = 0.92; Table 1).

As in the Manyu drainage basin, raw nodule prevalence was significantly higher in male (38.4%) than female (27.2%) subjects (P < 0.001, Table 2). However, the contrary was found with mf prevalence being higher for female (57.5%) than male (26.7%) subjects (P < 0.001, Table 2). Once more, sex did significantly not influence WMMfD (P = 0.364, Table 2). No major difference was observed between adjusted and raw prevalence (Table 3).

Like the case in the Manyu drainage basin, raw nodule prevalence was relatively lower in younger individuals (29.5.0%) than in adults (34.9%) with the difference not being significant (P = 0.102, Table 2). Raw mf prevalence was also similar in the two groups (P = 0.790). Similar results were found with adjusted prevalence (Table 4). However, the WMMfD was significantly higher for younger (5.04 mf/ss) than elderly (3.84 mf/ss) individuals (P < 0.001). The same trend was observed when taking into account different age groups with children (≤10 yrs) infected more frequently than elderly people (Table 2).

The results of *Simulium* dissection from the 2 catching points of the Mungo drainage basin are presented in Table 5. The number of L3 found in the head of dissected flies was 79.9 and 190 L3/1000 parous flies for the collection sites of Bolo and Bakumba respectively. These values were higher than the ones observed in the Manyu river basin.

The transmission potentials from the two collection points were high 282 and 1180.1 infective larvae/man/month for Bolo and Bakumba respectively while the infective rates were 2.7% and 6.0% at bolo and Bakumba respectively (Tables 5 and 6; Figure 5).

#### Meme drainage basin

The overall raw prevalence of nodule in this basin was 39.1% while that of mf was 52.7%. Both nodule and mf prevalence were the highest when compared to those of Manyu and Mungo river basins (P = 0.006 and P < 0.0001 respectively, Table 1). Adjusted prevalence gave a similar trend. Raw nodule prevalence varied from 13.6% to 67.6% (P < 0.001) and 15% to 77.4% with adjusted prevalence. Fifteen communities out of 16 had nodule prevalence greater than 20% among which 9 had nodule prevalence above 40% (Table 1 and Figure 2). Raw mf prevalence ranged from 21.2% to 91.1% (P < 0.001). This range relatively decreased with adjusted prevalence (28.6%-89.2%). Thirteen communities had a mf prevalence greater than 40% among which there were 3 with mf prevalence above 60% (Table 1 and Figure 3). The number of communities with mf prevalence above 60% increased from 3 to 5 after gender- and age adjustment. The overall CMFL (5.22 mf/ss) was higher than the ones found in the other drainage basins (P = 0.04). None of the communities had CMFL up to 10 mf/ss. CMFL ranged from 3.9 mf/ss to 8.7 mf/ss (P = 0.17), Figure 4, and Table 1.

Though nodule prevalence was higher in male (40.7%) than female (37.1%) subjects, sex did not significantly influence nodule and mf prevalence (P = 0.35, P = 0.92 respectively Tables 2 and 3). However, the infection was more intense in male (6.13 mf/ss) than female (4.57 mf/ss) subjects (P = 0.008, Table 2) like in the Manyu and Mungo drainage basin.

Despite the absence of children in the Meme river basin, younger individuals [15–29 years] were still found to have the highest mf prevalence and WMMfD compared to elderly ones (Table 2).

The results of *Simulium* dissection from the 3 catching points of the Meme drainage basin are presented in Table 5. The number of L3 found in the head of dissected flies was still very high; 271.2, 207 and 304 L3/1000 parous flies for the collection sites of Big Massaka, Bombele and Marumba I respectively.

The transmission potentials from the three collection points were also relatively high with 1211.7, 1128 and 90 infective larvae/man/month for Big Massaka, Bombele and Marumba I respectively. The same trend was seen with infective and infection rates (Tables 5 and 6; Figure 5). This river basin had the highest entomological indices.

#### Comparison between pre-control and present endemicity levels

Disease prevalence and entomological transmission indices generated were compared with pre-control data in those communities that were found to have historical data either published or unpublished [33-36]. Only adjusted disease prevalence was used.

In the Manyu drainage basin (North of South-West), the nodule prevalence at Nkonghaw (32.20%) and Ogurang (24.60%) were significantly lower than the pre-control levels of 62.50% and 80.30% respectively (P < 0.001; Table 7). In the Mungo and Meme drainage basins (South of South-West), nodule prevalence had significantly increased from pre-control levels of 19.66% (12.1-27.7%) to 53.2% (47.-60.6%) as presented in the Table 7. However, there was a marked reduction in mf prevalence when compared to historical data. The overall post-control mf prevalence in communities with historical data of 60.2% (49.2-74.0%) was significantly lower than the pre-control levels of 78.78% (58.8-98.1%) as depicted in Table 7. The same trend was observed for CMFL (Table 7) with a marked reduction from pre-control of 32.32 mf/ss (3.40-82.3 mf/ss) to 5.80 mf/ss (4.09-8.31 mf/ss). Although there was a relative reduction in Bolo, recent entomological indices (infective rate of 6.0% and monthly transmission potential of 1180 L3/man/month) were significantly higher than pre-control levels of 3.2% and 266 L3/man/month in Bakumba (Table 8).

**Table 7:** **Comparison of the pre-and post-control Mf, nodule prevalence and CMFL**

**Drainage basin**	**Villages**	**Pre-control Mf prevalence**	**Recent adjusted Mf prevalence**	**Pre-control nodule prevalence**	**Recent adjusted nodule prevalence**	**Pre-control CMFL**	**Recent CMFL**	**Authors of pre-control endemicity data**
**Mungo/Meme (South of South-West)**	Marumba I	63.0	73.7	16.5	51.1	4.79	6.13	Data collection (1991)
	Marumba II	58.6	52.2	12.1	47.0	3.40	4.17	Data collection (1991)
	Boa Bakundu	68.8	64.3	18.3	56.0	6.08	8.31	Data collection (1991)
	Bombanda	71.5	64.2	23.7	51.2	9.40	5.86	Data collection (1991)
	Bombele	79.1	74.0	27.7	60.6	10.24	5.21	Data collection (1991)
	Bakumba	95.3	50.7	-		82.3	6.76	Moyou *et al*., 1993 [36]
	Small Massaka	96.1	53.0			69.1	4.09	Moyou *et al*., 1993 [36]
	Bikoki	98.1	49.2			73.28	5.92	Moyou *et al*., 1993 [36]
	**Total**	**78.78**	**60.2**	**19.66**	**53.2**	**32.32**	**5.80**	
	Baduma,Bolo-Meboka,Weme and Kokaka community complex	87.0-97.1	46.1-62.6	60.6-80.7	42.5-58.9		3.27-4.64	Duke and Moore, 1966 [35]
**Manyu (North of South-West)**								Anderson *et al.,* 1974 [34]
	Mamfe (averall)	**96.8**	**40.2**	**80.7**	**32.6**			Anderson *et al*., 1974 [34]
	Nkonghau			62.50	32.20			Wanji et al. 2003 [25]
	Oguran			80.30	24.60			Wanji et al. 2003 [25]
	**Total**			**71.40**	**28.40**			

NB: Only communities with pre-control data were presented in this table; only adjusted recent prevalence presented.

Bold data represent mean parasitological indices.

**Table 8:** **Comparison between pre-control and post-control monthly biting rates, infective rates and monthly transmission potentials at different sites in the study area**

**Drainage basin**	**Capture site**	**Monthly biting rate (MBR) pre-control**	**Monthly biting rate (MBR) after 12 yrs of treatment**	**Infective rate (%) pre-control**	**Infective rate (%) after 12 yrs of treatment**	**Infection rate (%) pre-control**	**Infection rate (%) after 12 yrs of treatment**	**MTP pre-control**	**MTP after 12 yrs of treatment**	**Authors of pre-control data**
**Mungo/Meme (South of South-West)**	Bolo	11122	12636	3.1	2.7	12.8	8.3	570	282	Enyong *et al.*, 2006 [37]
	Bakumba	9374	24408	3.2	6.0	7.5	10.8	266	1180	Moyou *et al*., 1993 [36] and Enyong *et al.*, 2006 [37]

NB: Only communities with pre-control data were presented in this table.

MBR (Flies/man/month) = Monthly biting rate.

MTP (L3/man/month) = Monthly transmission potential.

## Discussion

In this study, we assessed the parasitological status and the entomological indices in three drainage basins in the rain forest area of Cameroon with a decade of ivermectin treatment. We observed a reduction in mf prevalence and intensities from pre-control levels with the reduction being more pronounced in CMFL. However, there was no comparable drop in entomological indices. Our results indicate that onchocerciasis is still meso and hyper endemic in the three drainage basins. In the forest area, besides very high pre-treatment ATP as described by Duke, the availability of fast flowing rivers contributes to the development of the large number of black flies necessary for efficient transmission and this could support these findings. There were remarkable variations between the three river drainage basins with the parasitological and entomological indices being higher in the South of South-West (Mungo and Meme). This observation could be explained by the fact that in the Manyu river basin, individuals stay far from streams and the existence of community pipe-borne water keeps them away from blackfly breeding sites. Moreover, in the Manyu, the communities were situated in the valleys where river flow rates are lower whereas in the Mungo and Meme they were found on the slopes (Figure 1) where river flow rates are high and favour maximum *Simulium* breeding. The intensity of infection was still high in the 3 drainage basins in younger individuals including children less than 10 years who were born after the launch of the control programme with ivermectin. These results are indicative of continued transmission. This means onchocerciasis elimination cannot yet be envisaged in this rain forest of Cameroon. The study was carried out in July when most children had gone for holidays and the few remaining were not all willing to take part in the study hence the absence of children in the Meme river basin.

Out of the seven sites where we collected entomological data, baseline data existed only for the Bolo and Bakumba sites (Mungo river basin). Comparing our results to those of Duke *et al.* [35], there was a remarkable drop in the infection rate (all larval stages), the L3/infective fly, while the infective rate remained the same. Based on these results, it can be said that the drop in the CMFL did not necessarily translate into a significant reduction in the above-mentioned entomological indices. This may also explain the relatively high mf prevalence obtained in most of the communities surveyed globally. The same trend was seen while comparing our entomological data in Bolo with that collected in 1998 and 1999 [37]. Moreover, in Bakumba we noted that the pre-control infective rate was lower than the infective rate observed in this study. However, there was an increase in MTP when compared to those from previous studies [21] and this tendency was reflected in the mf and nodule prevalence of most of the communities surveyed in the Mungo drainage basin.

Although previous studies carried out in Mali and Senegal [10] purport to demonstrate the feasibility of onchocerciasis elimination with ivermectin in endemic areas, it should be pointed out that those studies were conducted in savannah regions where the dry season lasts for about 9 months. Optimum breeding conditions for the *Simulium* larvae, i.e. availability of turbulence and suitable support, are found periodically in the year in the large perennially-flowing rivers or in the rainy season tributaries [38]. This negatively affects the transmission of the disease (scarcity of blackflies) and by so doing favours disease elimination.

The situation is different in rain forest areas; where optimum breeding conditions for *Simulium* larvae are found almost throughout the year and hence the perennial nature of breeding sites. The hilly nature of the study area in particular, contributes to the creation and maintenance of appropriate river flow rates for the establishment of permanent *Simulium* breeding sites hence, continuous transmission. In addition, there are a greater number of *Simulium* species with different vectorial capacities in the rain forest compared to Savannah zones [39]*.* The transmission potential (the number of potentially infected flies available for transmission to the human host per unit time) of forest vectors is known to be higher than that of their savannah counterparts [40]. Using cytotaxonomic techniques, five *simuliid* species were found to transmit *O. volvulus* in Cameroon [41,42] *S. damnosum s.s.*, S. *sirbanum, S. mengense S. yahense* and *S. squamosum* A, B and C*. Simulium damnosum s.s*. and *S. sirbanum* are common in the savannah zone whereas *S. mengense* is present both in forest and savannah (but restricted to rivers showing great turbulence or waterfalls). In our study site, S*. damnosum* s.s. and *S. squamosum A and C* have been reported [21,22,43,44]. *S. damnosum* s.s. seems to be more adapted to the forest environment as well as the forest parasites (mfs) as demonstrated by previous studies and described as a notion of “well-adapted” *Onchocerca–Simulium* complexes [35,45].

Interestingly, all these reported species (*S. damnosum* s.s. and *S. squamosum A and C*) have been shown to exhibit the phenomenon of “limitation” previously described as one of the factors favouring the transmission of the disease [45-49]. “Limitation” describes the situation where vectors are efficient even at very low parasite densities [50], the greater the number of microfilaria ingested, the smaller the percentage of them that reach the haemocoele [51]. Infected flies with low mf load have a greater chance of survival and maintaining transmission of the parasites [48,52]. The peritrophic membrane has been shown to be responsible for the limitation phenomenon, reducing the number of ingested mfs reaching the haemocoele. Although this phenomenon is observed both in the savannah and the rain forest areas, the percentage reduction has been demonstrated to be smaller in the rain forest [53,54] compared to the savannah areas [46]. This means that the *Simulium* species found in the forest areas have the capacity to transmit more infective larvae than their savannah counterparts. This could also explain the high transmission indices obtained in this study. Moreover, the African forest *O. volvulus* strain has been demonstrated to have lower *Wolbachia* levels [55-57] and as such elicits weaker anti-bacterial responses within simuliids, hence the higher larval loads observed in *Onchocerca-Simulium* forest combinations [58]. This might also explain the high parasitological indices observed in the present study, although more investigations are needed to confirm this assertion. In such circumstances mass drug treatment alone would not interrupt transmission [59]. Control may only be achieved using a combination of strategies (Mass drug distribution including use of a macrofilaricide, vector control and vaccination, when this becomes available as suggested by De Souza and others) [59].

In our study after 10 to 12 years of treatment, the number of L3 in head/1000 parous flies fluctuated between 30 in Barumba and 271 in Big Massaka (Table 5), which are 60 and 542 times above the predicted threshold of 0.5/1000 parous flies (0.05%) used by Diawara and others [10] and recommended by APOC. This could again be explained by the fact that the pre-control endemicity was very high in the rain forest in addition to the above-mentioned factors such as the abundance of the vector, more efficient vectorial capacity, presence of breeding sites, limitation phenomenon exhibited etc. all of which contribute to the establishment of high transmission indices in the forest areas. As such, more time will likely be required to interrupt onchocerciasis transmission through CDTI [60].

Entomological investigations carried out in four Sudan-Savannah villages in West Africa indicated pre-control Annual Transmission Potentials (ATP) varying between 500–19000 infective larvae/man/year [38,61-64]. These values were far lower than those (897–87846 infective larvae/man/year) observed in a similar study in rain forest villages in the South West of Cameroon [43]. In that study, there was a positive association between the intensity of infection in the human populations and the transmission potential. Duke demonstrated in the same study that the transmission potential in the forest zone depends mainly on the absolute numbers of flies biting and to a lesser extent on the minimal microfilarial reservoir available for those flies. In many places in the forest zone, reservoir levels might be far in excess of the minimum required to maintain the parasite as a significant pathogen in the community [65]. However, it should also be noted that entomological transmission indices can vary dramatically from one year to another, because of differences in rainfall and other climatic phenomena [66].

Our entomological study did not use molecular assays to determine whether all L3 larvae were *O. volvulus,* or included some animal *Onchocerca* species. Nevertheless, OCP data make it possible to specify the vectorial role of the various species of the *Simulium damnosum* complex in the various ecological zones. A study of the parasites recovered from *S. damnosum* revealed that parasites other than *O. volvulus* were so rare as to be negligible [43]. In forest zones the contribution of animal parasites to *S. squamosum* infectivity rate is 3% [67]. Deducting 3% from the L3 larvae collected at our various study sites would not make much difference to the infective rates and MTPs observed. Moreover, the high mf prevalence and intensity of infections in children clearly indicate that the vast majority of the L3 larvae carried by the blackflies in the study areas, which contain no ranches or game reserves, could be *O. volvulus*.

Of all the 39 surveyed communities in our study, 23 (about 60%) communities (among which 13 from the Meme river basin) still had raw mf prevalence above 40% and 35 (about 90%) with raw nodule prevalence above 20% among which 16 (41%) with greater than 40%. With the ONCHOSIM prediction model for elimination of onchocerciasis, it is anticipated that with a pre-control endemicity level of about 70 mf/ss, which corresponds to around 80-100% mf prevalence, 10 years of ivermectin treatment with 70% treatment coverage will be necessary to bring the mf prevalence to at least 40% [60]. The results obtained in this study do not support such a predicted trend. The prevalence in the majority of our communities is still far above 40%, which in turn remains very far from the threshold for elimination recommended by APOC (less than 5% prevalence in all surveyed communities and less than 1% in 90% of surveyed communities). Most of our surveyed communities had pre-control CMLF less than 70 mf/ss, one could have expected the level of endemicity after more than a decade of CDTI to be much more reduced than what is observed.

This high prevalence could also be due to poor compliance with ivermectin treatment [68]. The low compliance could have resulted from fear of severe adverse events (SAEs) caused by the drug. Besides itching, it has been shown that people co-infected with *Loa loa* and having high *Loa loa* mf loads develop encephalopathy following ivermectin treatment [69-73]. Indeed, cases of SAEs were reported at the onset of mass ivermectin administration in this study area [17]. These could also explain the low therapeutic coverages (<45%) registered in the area at the beginning of the CDTI programme (1999–2002). Despite the sharp increase in therapeutic coverage from 2003 following remedial efforts to allay fears of SAEs, a report of one of the CDTI Projects in the South West of Cameroon [17] still revealed a high rate of refusals (27.7%) among those eligible for treatment. This high refusal rate was shown to be closely linked to a high level of skepticism, doubt and pessimism among community members. Likewise, it was found that, in general, communities did not have enough information on side effects to allay their fears in an evaluation of the implementation of TCC9/Mectizan Expert Committee guidelines in areas of Cameroon co-endemic for onchocerciasis and loiasis [74]. Though precautions were taken to remedy the situation, the results of the present study suggest that people in the South West Region remained skeptical and reluctant to take the drug [65]. This could also explain the increase in both parasitological status and entomological indices observed in some communities in the Mungo/Meme drainage basin. However, to clarify this situation, investigations aiming at establishing a relationship between compliance and parasitological indices in humans are necessary.

Generally, in this study we observed that the burden of onchocerciasis as measured by the mf load has been reduced after 10 to 12 years of repeated annual treatment with ivermectin. However, the reduction is far from the point where the disease can no longer be considered as a public health problem. The results also indicate that reducing the prevalence, intensity and transmission of *O. volvulus* infection below the threshold level of elimination in this study area will take a much longer time. Distributing ivermectin twice a year with at least 85% therapeutic coverage may reduce the time required to reach the threshold for elimination as shown by ONCHOSIM [60]. Work done by Cupp and Cupp [75] in Guatemala suggested that twice yearly treatment, covering all eligible persons could interrupt transmission of onchocerciasis without vector control measures. However, the fear of SAEs following ivermectin treatment remains a very serious and perhaps insurmountable impediment to successful control in the short term. It is worth mentioning that in the Americas ivermectin is not distributed using the CDTI strategy.

The question of resistance to ivermectin can no longer be ignored. The intensive and widespread use of ivermectin will eventually lead to development of resistance [76-82]. Furthermore, the high levels of transmission in forest zones suggest that once resistance is established, it could rapidly spread.

Over the past few years, increased attention has been paid to the obligatory symbiotic relationship between some filarial nematodes and *Wolbachia* endobacteria [83,84]. This has been exploited to demonstrate the inhibitory effects of anti-*wolbachia* drugs on filarial embryogenesis leading probably to permanent sterilization of the female filariae [85]. In addition to inhibition of embryogenesis, repeated oxytetracycline treatments for several months resulted in the complete disappearance of adult worm nodules [86]. Similar results have been obtained with other members of the tetracycline family such as doxycycline [87,88]. These drugs have no or little effect on *Loa loa* whose rapid killing by ivermectin triggers SAEs in individuals with heavy *Loa loa* microfilarial loads [89]. With appropriate community and socio-anthropological support, anti-*Wolbachia* drugs could constitute a valuable supplement to ivermectin in such areas to treat infected individuals who are reluctant to take ivermectin because of fear of SAEs using test and treat or test and not treat strategy [90-92].

## Conclusions

This study has demonstrated a dramatic reduction in CMFL in three river basins after 10 to 12 years of ivermectin treatment although these changes were not reflected by comparable reduction in entomological indices and onchocerciasis prevalence. The parasitological and entomological findings in the three river basins allow us to conclude that onchocerciasis transmission is still on-going in rain forest communities of south west Cameroon where onchocerciasis and loiasis are co-endemic. The transmission seems to be more prominent in the Mungo and Meme River basin where *Simulium* breeding conditions are more favourable, due probably to the geography and topography of the terrain as well as the location of the communities with respect to rivers. Based on these findings, we conclude that some forest communities in Cameroon are far from satisfying the WHO (2001) guidelines [93] for onchocerciasis elimination. It would be interesting to further investigate the reasons of this persistent transmission of onchocerciasis in the areas despite over a decade of control efforts using ivermectin; such investigations should establish the relationship between compliance to ivermectin treatment and the parasitological indices in humans. Alternative strategies for the control and eventually elimination of onchocerciasis should be envisaged in the rain forest areas where *L. loa* co-exist with Onchocerciasis or Lymphatic filariasis.

## Additional file

Additional file 1: Figure S1. Study design.

## Abbreviations

OCP: Onchocerciasis Control Programme
APOC: African Programme for Onchocerciasis Control
CMFL: Community Mf Load
WMMfD: Williams Mean Mf Density
MF: Microfilaria
ONCHOSIM: Onchocerciasis transmission elimination simulation model
CDTI: Community-directed treatment with ivermectin
CBTI: Clinic-Based Treatment with Ivermectin
SAEs: Severe adverse effects
MEC: Mectizan® Expert Committee
TCC: Technical Consultative Committee
REA: Rapid epidemiological assessment
L1: Onchocerca *volvulus* larval stage 1
L2: Onchocerca *volvulus* larval stage 2
L3: Onchocerca *volvulus* larval stage 3
MBR: Monthly biting rate
DBR: Daily biting rate
MTP: Monthly transmission potential
RAPLOA: Rapid Assessment Procedure for Loaisis
