# Supplementary figures and images for: Situation analysis of parasitological and entomological indices of onchocerciasis transmission in three drainage basins of the rain forest of South West Cameroon after a decade of ivermectin treatment
Source: Parasit Vectors. 2015 Apr 2;8:202. doi: 10.1186/s13071-015-0817-2 (PMC4393872; doi:10.1186/s13071-015-0817-2)

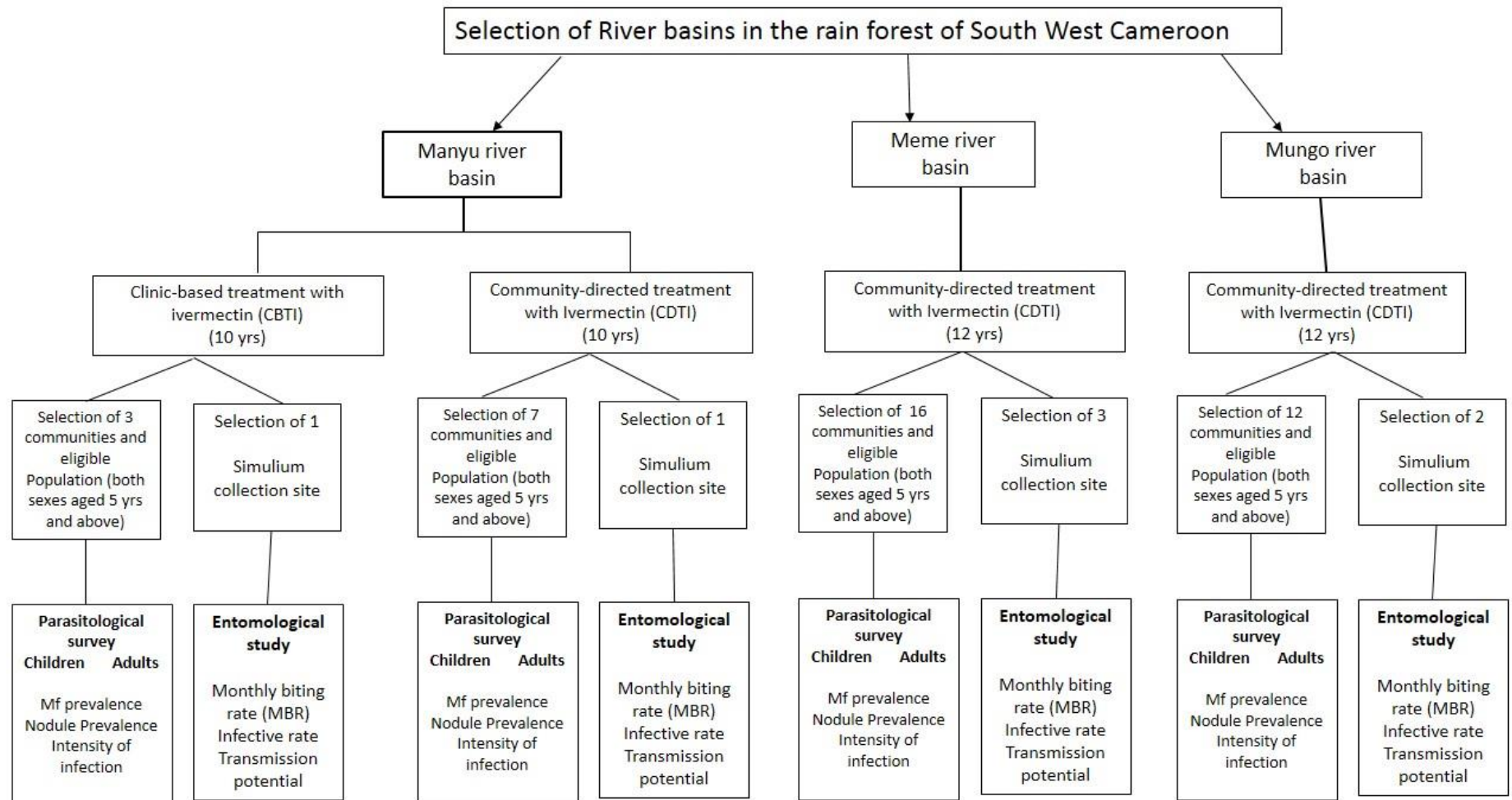

**Figure S1:** Study design

Supplement: Additional file 1: Figure S1. — Study design. [file 13071_2015_817_MOESM1_ESM.pdf]
